# Multi-physiology modeling of the immune system in the era of precision immunotherapy

**DOI:** 10.3389/fimmu.2025.1548768

**Published:** 2025-05-29

**Authors:** Sunghyun Hong, Kyemyung Park

**Affiliations:** ^1^ Department of Biomedical Engineering, College of Information and Biotechnology, Ulsan National Institute of Science and Technology (UNIST), Ulsan, Republic of Korea; ^2^ Graduate School of Health Science and Technology, College of Information and Biotechnology, Ulsan National Institute of Science and Technology (UNIST), Ulsan, Republic of Korea

**Keywords:** multi-physiology modeling, systems immunology, precision immunotherapy, multi-omics data, multiscale modeling, quantitative systems pharmacology

## Abstract

Immunotherapies using new modalities, including antibody-based drugs, nanoparticle-delivered drugs, and adoptive cell therapy, have become major treatment options for immune-related diseases such as cancer, autoimmune diseases, and infections. Although data characterizing individual patients’ pharmacological responses, immune statuses, and clinical outcomes become increasingly available, predicting individual patients’ immunotherapeutic responses for developing and deploying optimal immunotherapies remains challenging. Here, we propose “multi-physiology modeling” of the immune system that integrates omics-based and dynamic systems modeling-based systems immunology and pharmacometrics modeling on top of basic and clinical immunology. The multi-physiology modeling approach aims to integrate different physiological systems to realistically simulate the multi-scale and complex interactions of the immune system under intervention by immunotherapeutic agents for predictive immunotherapies tailored to individual patients. This will accelerate not only our understanding of basic immunology related to immune-related diseases but also the efficiency and accuracy of clinical immunotherapeutics in the era of precision immunotherapy.

## 1 Introduction

Throughout the last decade, new therapeutic modalities collectively called immunotherapies have emerged as major medical practices for curing or preventing immune-related diseases such as cancer, autoimmunity, and infection (1–3). Although immunotherapies have shown great potential to cure such diseases, the lack of reliable predictive ability for individual patients’ therapeutic responses still needs to be overcome (4). Personalized and precision medicine aims to predictive therapies that proactively adjust treatment plans by predicting individual patients’ responses or side effects to treatment before or during the treatment. This will enable the delivery of an optimal drug or a combination of drugs to appropriate patients at precise dosages and timings (5–7). Implementing this promising framework in immunotherapy requires the accurate characterization of the pharmacologic behaviors of immunotherapeutic agents, the baseline and therapy-induced changes of immune statuses, and the resultant clinical outcomes in detail for individual patients. Recent advances in new omics technologies, data science, and computational science have made it possible to work with biological and clinical data at a higher resolution than ever before. All this information should be transformed into prediction models of therapeutic responses tailored to individual patients’ personalized course of treatment (8). As personalized and precision medicine in immunotherapy becomes a near reality, more patients would likely benefit from immunotherapy (9).

To advance toward this promise, the prediction models should simultaneously describe quantitative pharmacometrics behaviors of immunotherapeutic agents and intricate immune behaviors while addressing inter-individual heterogeneities in such behaviors in a single framework. However, achieving such a framework has been staggering mainly due to separate pursuits for these aspects by experts from respective fields, needing more communication across those communities. For instance, immune behaviors are coordinated via sophisticated networks of interactions between numerous cellular and molecular components. These immune networks are intertwined with feedback and feedforward loops across scales spanning from intracellular and cellular to the organismal levels, resulting in nonlinear behavior that contributes to the lack of predictability (10–14). Although so-called systems biological approaches tackle such a complexity of the immune system, a considerable dichotomy between omics-based and dynamic systems modeling-based approaches hinders a realistic description of the immune system as prediction models. Omics data-driven analyses using statistical or machine learning approaches effectively uncover patterns directly from existing high-throughput datasets. However, purely data-driven predictions remain inherently limited by the availability and completeness of data, as they rely on interpolation within the bounds of observed clinical scenarios from which data are obtained. In contrast, dynamical systems modeling approaches integrate mechanistic immunological knowledge that potentially enables predictions even in clinically unexplored contexts through their capacity for extrapolation beyond existing data. However, mechanistic modeling is limited by its tendency to describe the system rather simplistically. From a different route, population pharmacometrics, including pharmacokinetics(PK) and pharmacodynamics(PD) modeling, utilizes mathematical modeling to provide quantitative information for dose-concentration-efficacy/toxicity relationships and, therefore, is instrumental in drug development, clinical trial design, and treatment strategies (15–17). Quantitative systems pharmacology (QSP) has been extending its boundary to integrate more biological pictures related to drug response (18–20). However, due to its origin in modeling the system as well-mixed compartments using ordinary differential equations (ODE), what QSP promises remains limited in capturing realistic immune behaviors, such as the heterogeneity of single cells along the spatial and phenotypic axes.

Here, to overcome existing limitations for establishing personalized and precision immunotherapy based on prediction models, we propose an overarching umbrella, “multi-physiology modeling” of the immune system as a common goal, toward which collective efforts are needed to concretize this conceptual framework. This framework should encompass population pharmacometrics and its extension to QSP, omics-driven and dynamical systems modeling-driven systems biology, and basic and clinical immunology on equal footing. We hope to overcome prejudices residing in each field via close communication across fields to identify impending problems to be solved to achieve the multi-physiology modeling of the immune system. In the following sections, we review each of the relevant fields and discuss their limitations. Then, we introduce a conceptual sketch of the multi-physiology modeling of the immune system, followed by a discussion on its promises.

## 2 Currently available immunotherapeutic modalities

Immunotherapeutic modalities directly targeting immune system components include antibody-based drugs, nanoparticle-delivered drugs such as mRNA vaccines, and adoptive cell therapies (1, 3, 21, 22). Antibody-based drugs utilize monoclonal antibodies designed to bind to target proteins on immune cells, allowing for precise control of the immune response. These antibodies can enhance antitumor activity against cancer or reduce excessive immune responses in autoimmune diseases. One significant application of monoclonal antibodies is as checkpoint inhibitors targeting immune checkpoints such as PD-1/PD-L1 and CTLA-4 to reinvigorate T cell cytotoxicity against cancer cells (23–25). Additionally, monoclonal antibodies treat chronic inflammatory diseases by targeting cytokines (26). Nanoparticle-based delivery systems can directly modulate immune system behavior by intracellular targeting (27). This approach has revolutionized vaccination, as demonstrated by the rapid development and high efficacy of COVID-19 vaccines (28, 29). These vaccines use lipid nanoparticles to deliver mRNA into cells, translating it into viral proteins that stimulate an immune response without causing disease. Nanoparticles protect mRNA from degradation and facilitate its delivery to target cells, ensuring efficient uptake and protein production (27–29). This technology holds promise for treating various diseases, including cancer and genetic disorders, by enabling precise delivery of therapeutic mRNA to specific tissues (30, 31). Adoptive cell therapy manipulates patients’ immune cells to improve the treatment of diseases. This therapy involves the isolation of immune cells, such as T-cells or natural killer (NK) cells, from a patient, engineering or multiplying them to boost their disease-fighting abilities, and reintroducing them into the patient (32–34). For example, in CAR-T cell therapy, T-cells are altered to express chimeric antigen receptors to target cancer cells (34–36). Adoptive cell therapy is not limited to cancer treatment. It is also being investigated for autoimmune diseases and infectious diseases. For instance, regulatory T-cells (Tregs) can be expanded to suppress excessive immune responses in autoimmune conditions (37, 38). These immunotherapies can treat previously intractable diseases such as cancer and autoimmune diseases and respond promptly to emerging pandemics. However, although various options for immunotherapies are available, they vary in efficacy between individuals, and it is difficult to prescribe the optimal dosage (39–41). In this regard, the related and optimized pharmacometrics modeling is essential.

## 3 PK/PD modeling in new emerging therapeutic modalities and its limitations

The PK/PD models have provided a robust quantitative basis for assessing the drug’s pharmacometric properties (15, 16). The PK model describes the drug’s absorption, distribution, metabolism, and excretion (ADME) and changes in drug concentration over time. The PD model explains the physiological or pharmacological responses induced by the drug concentration in the body. Furthermore, to capture inter-individual variabilities and their correlates, such as age, gender, or genetics, nonlinear mixed-effect modeling (NLME) is used. NLME includes fixed and random-effect parameters. Fixed-effects parameters represent the tendencies across the entire population. Random-effect parameters account for individual variations of fixed-effect parameters and are further modeled to be linked to covariates (17). The PK/PD models are constructed as simplistic representations, treating bodies or organs as homogeneous compartments analogous to well-mixed containers. These models could describe the quantitative pharmacologic behavior of antibody-based drugs (42–46), nanoparticle-delivery-based therapies (including mRNA vaccines) (47–50), and adoptive cell therapies (7, 51–55). However, such a simplistic way of describing the system is unsuitable for incorporating complex immunological processes, thereby rendering immunological complexity linked to modern immunotherapies not fully captured by existing PK/PD models.

A newly emerging field, quantitative systems pharmacology (QSP), has addressed some challenges by incorporating more mechanistic mathematical immune system models into pharmacometric models. Significant efforts have been made in compiling existing mathematical models of immune behaviors in various disease contexts, suggesting a new direction of incorporating newly uncovered immune features from new data types, and applying those models in accelerating drug development and target identification that grows with the vast combinatorial search space of combination therapies (56–58). For example, Arulraj et al. (59)demonstrated that a QSP model of triple-negative breast cancer augmented with bulk tumor data could be utilized to perform in silico (virtual) clinical trials and identify unrecognized biomarkers linked to therapeutic outcomes of anti-PD-1 therapy. There are also similar endeavors in adaptive cell therapy and mRNA vaccination in the QSP framework (49, 54, 60).

Although QSP foresees a future of model-informed drug development and personalized and precision immunotherapy, those employing the QSP framework still need more detailed descriptions of the immune system. For instance, the recent literature on immune diseases reveals highly heterogeneous single cells dispersed throughout the space with complex interactions among those (61). Moreover, immune behavior tends to be driven not by immune cells with major phenotypes but by the minorities of those heterogeneously dispersed in cellular phenotypic space (62, 63). Therefore, the inherent language of QSP, employing the picture of the immune system with merely “more” compartmentalization using ODE, may not be suitable. Hence, the continuing effort of the current practice of QSP may not achieve what it promises. To this end, we previously demonstrated that hybrid modeling capturing the multiscale nature with a continuum of phenotypic space among even the same cell type can give rise to non-intuitive immune behavior for establishing or breaking immune homeostasis (10, 62, 64).

Taken together, the difficulty of predictively modeling immunotherapeutic responses is a multifaceted problem rooted in the complex nature of the immune system and the insufficiency of reliable biomarkers due to the sparse characterization of the system (65–68). To address this, we need more comprehensive immune profiling together with methodologies to transform such profiling into prediction models that capture complex immune behaviors.

## 4 Dichotomic systems immunological approaches and their reconciliation needed

Systems immunology has emerged as a field that simultaneously considers many molecular and cellular constituents of the immune system quantitatively to provide holistic and predictive views of how the immune system operates (62, 69–71). Systems immunology possesses a dichotomy of being based on either high-throughput omics data or dynamical systems modeling.

Single-cell and spatial omics technologies have become a routine driven by technological advancements and the increasing need to comprehensively understand cellular heterogeneities and functions and their relations to immune regulation (72–74). Single-cell RNA sequencing (scRNA-seq) profiles transcriptomes at the single-cell level, which provides granular insights into cell types, states, and their roles in various immunological processes (75–78). In addition, to capture additional layers of cellular functions and regulatory mechanisms, researchers have developed methods to profile proteomes, epigenomes, and spatial information in single cells (73, 79–82). Multi-omics approaches provide a more holistic view of cellular phenotypes, combining the strengths of each modality to reveal more profound insights into cell biology. For example, CITE-seq (Cellular Indexing of Transcriptomes and Epitopes by sequencing) allows simultaneous measurement of mRNA and surface protein expression in the same cells, bridging the gap between gene expression and functional protein data (83, 84). Moreover, integrating scRNA-seq with ATAC-seq (Assay for Transposase-Accessible Chromatin using sequencing) has opened new avenues for understanding the upstream regulatory landscapes, such as enhancers and promoters that control gene expressions (85, 86). Spatial omics is another critical development in this field, preserving the spatial context of cells within tissues. This technology enables researchers to study how cells are organized and interact within their native microenvironments (87–89).

Dynamic systems modeling-based systems immunology utilizes many types of mathematical modeling to provide unique and nonintuitive insights into immune dynamics (70, 90–93). One primary type is the ordinary differential equation (ODE) model, which describes the interactions between immune cells, pathogens, and signaling molecules over time at cellular or molecular population levels. Such models can capture the time-dependent rates of changes in the quantities (the numbers or densities of cells or molecules) associated with each component (94–96). For example, ODE models can describe temporal changes in population sizes of immune cells and pathogens based on their growth, death rates, and interactions among them (97–99). Partial differential equations (PDE) are another essential modeling tool for the immune system, well suited to modeling spatial changes in the immune system over time. For example, a PDE model can describe the interaction of immune cells and pathogens as infection spreads within a tissue. The model can represent the spread of pathogens and the subsequent response of immune cells as the temporal evolution of spatial distributions of cells, pathogens, and/or signaling molecules’ concentrations (100, 101). Beyond these deterministic methods, there are also methods to capture the inherent uncertainty and variability of living phenomena. Stochastic models incorporate random elements for temporal fluctuations in immune cell counts, cytokine levels, or intracellular molecular copy numbers (102, 103). This approach helps to understand the dynamics of immunological/biological processes occurring with small cellular or molecular populations and helps predict the probabilistic outcome of immunological processes. Agent-based models (ABMs) are organized differently from the above approaches. Such models simulate the behavior and interactions of individual agents, such as cells. They can capture collective behavior shaped by interactions between individual agents by imposing migration patterns and interaction rules of immune cells (104, 105). For example, inflammatory responses at the site of infection can be modeled using agent-based modeling with the interactions of individual cells as simple discrete rules or ODEs (106, 107). More detailed reviews and analytical tools of various modeling approaches can be found in references (108–120).

Although both omics data-driven and dynamical systems modeling-driven approaches are legitimate in a quantitative and holistic understanding of the immune system, seamless integration of these is needed to accelerate capturing the genuinely complex and dynamic picture of the immune system. We identify two general challenges in this regard. First, although more comprehensive technologies to profile the immune systems are rapidly emerging, mainstream practice in the omics field is still in the descriptive cataloging of numerous cellular and molecular components. Transforming such comprehensive information about the immune system into predictive models of dynamic immune behavior is still in its infancy (69). Second, although various mathematical tools have been developed to model the immune system, each has its scope confined within particular biological layers simplistically. We need an overarching mathematical/computational framework for integrating each tool to describe immune behavior occurring across multiple biological layers. Given that we have successfully integrated the dichotomic systems immunological approaches, this should eventually be translated into therapy, seamlessly integrating the population PK/PD modeling framework and flexibly adapting to various immunotherapeutic modalities.

## 5 Toward multi-physiology models of the immune system: synergy of PK/PD modeling frameworks and systems immunological modeling beyond QSP

Thus far, we have dealt with the challenge in predictive immunotherapies arising due to the insufficiency of existing PK/PD modeling and the infancy of “genuine” systems immunological modeling. To achieve better predictions of the immunotherapeutic responses, therapeutic target identification, and designing therapeutic regimens to provide each individual patient with a better cure, we need to demonstrate the complex immune behavior realistically as in silico models. Here, “realistic” models should encompass cellular and molecular players that interact together across multiple layers of biological organizations. This multiscale nature of the immune system gives rise to non-intuitive and nonlinear behavior across space and time, in contrast with the models with oversimplification as the most existing mechanistic immune models.

**Figure 1:**
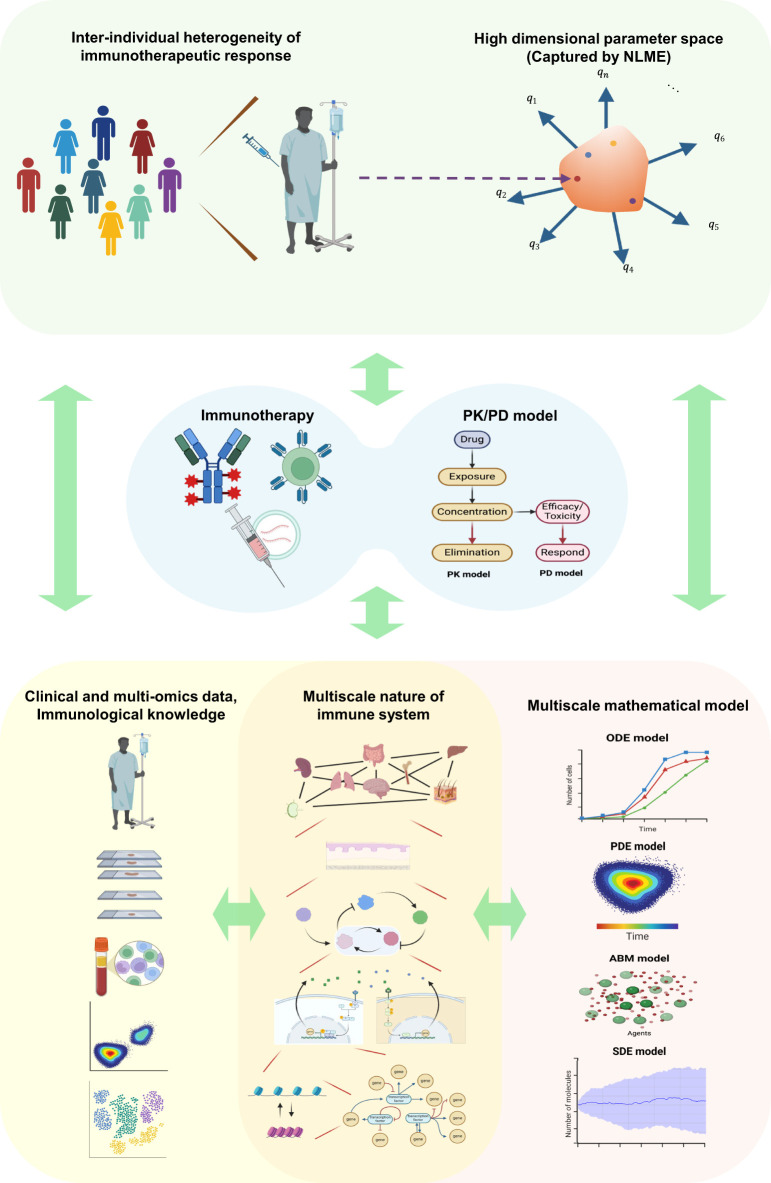
Schematic of the multi-physiology modeling framework. The inter-individual heterogeneity of patients’ immune statuses and immunotherapeutic responses is represented as points within a high-dimensional parameter space captured by NLME. Parameters and variables derived from integrated multi-omics and clinical data and immunological knowledge are utilized to construct an integrated in silico model that combines PK/PD modeling and multiscale mathematical modeling. The model’s outputs can guide immunotherapy strategies at the individual patient level. Throughout treatment, continuous immune profiling of individual patients can update immunotherapy strategies in a model-informed manner, enabling personalized precision immunotherapy. NLME, Nonlinear mixed-effect modeling; ODE, ordinary differential equation; PDE, partial differential equation; SDE, stochastic differential equation; ABM, agent-based model; Created with BioRender.com.

Here, we propose an overarching umbrella, “multi-physiology modeling” of the immune system (**Figure 1**). The expression “multi-physiology” is analogous to “multi-physics” in engineering and earth science fields, where different aspects of systems are modeled simultaneously (121). We regard this as a central ground, treating all relevant fields equally rather than emphasizing one and extending to others. In this approach, we aim to realistically describe the immune system in silico exactly how it operates across multiple spatiotemporal scales with many constituent components interacting. In addition, we seamlessly integrate these immune models with pharmacometric frameworks that interface with immunotherapeutic agents and patient responses with inter-individual variability. This framework should be flexible enough to be continuously updated by newly accumulating knowledge in the relevant immune systems and diseases accelerated by quantitative omics data and be easily deployed in immunotherapy by accounting for unique pharmacological behaviors of novel and emerging immunotherapeutic agents.

**Figure 2:**
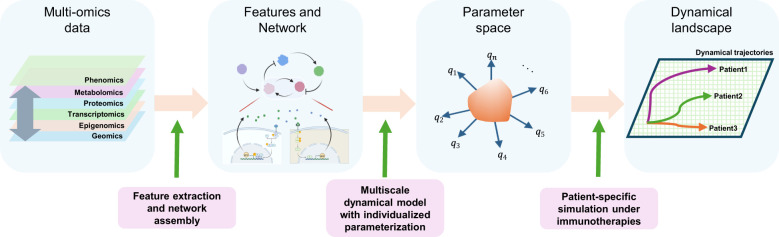
Construction of a multi-physiology model. Features are extracted from multi-omics data from patients, such as cellular features (such as abundances or locations) or intracellular features (such as molecular levels or signaling activation statuses), and assembled as multiscale networks using various computational tools. These networks form the basis for multiscale dynamical models with individualized parametrizations across a high-dimensional parameter space, using NLME and “pre-training”. A dynamic landscape is explored using a multi-physiology model across the high dimensional parameter space under immunotherapies, followed by patient-wise predictions.

To realize this, we should assess missing elements for methodological breakthroughs to establish multi-physiology models (122). An urgent need in mathematical modeling is to develop mathematical/computational frameworks to describe the multiscale spatio-temporal nature of the immune system. These frameworks need to seamlessly and flexibly integrate various modeling methods, such as ODE, PDE, SDE, or agent-based modeling, that tend to be independently used for their respective target layers of biological organizations. In addition, such frameworks should be able to faithfully encompass realistic immunologic pictures based on prior knowledge, experimental literature, and, nowadays, quantitative single-cell and spatial multi-omics data (**Figure 2**). We envision that this can be achieved, first, by extracting relevant multiscale and dynamic immunological features quantitatively from such dispersed sources. Quantitative features include cellular features such as cell-type annotations and spatial locations (if available through imaging-based data), and subcellular statuses such as molecular abundances and functional signatures. Then, we assemble those as networks of features interacting across scales, encompassing intercellular and intracellular connections. We can utilize tools such as CellPhoneDB (123), CellChat (124), and LIANA (125) to infer cell–cell communications through ligand-receptor pairs. OmniPath (126) can be used to reconstruct signaling networks. SCENIC+ (127) and CollecTRI (128) support the inference of gene regulatory networks, and NicheNet (129) provides multi-layered communication inferences. These tools operate based on curated knowledge-based databases, which are continuously expanded under various cell-type-specific perturbation conditions (72, 130, 131). Finally, we translate the network scaffolds into mathematical/computational (or dynamical) models by constructing reaction networks with propensity functions that define rates for each reaction. A major hurdle to be overcome is the general trade-off between data throughput and temporal resolution in available data. High-throughput data often sacrifices temporal details, making it difficult to extract dynamic patterns while maintaining high-dimensional biological complexity. For example, single-cell RNA sequencing or spatial transcriptomics data allow detailed snapshots of cellular states across thousands of cells but are typically limited to a single or a few time points due to cost and technical constraints. In contrast, blood-based biomarkers can be collected repeatedly, allowing immune responses to be tracked over time with lower throughput. Given that we have established all these, we should be able to intuitively interpret the multi-physiology models as we would analyze a much simpler model with a few variables and parameters by overcoming the difficulty in handling the inevitable high-dimensional parameters and variables in the models (132).

Ultimately, the multi-physiology modeling should embrace inter-individual heterogeneities of immunological processes and immunotherapeutic responses through individualized model parametrizations and initializations (**Figures 1** and **2**). NLME has been crucial for parametrizing population PK/PD models accounting for inter-individual variabilities. Since NLME mainly deals with ODE-based compartmental models, further methodological developments are needed to apply it in multi-physiology modeling. One likely barrier to establishing NLME in the multi-physiology modeling of the immune system is the obsession with measuring and identifying (or fitting) high-dimensional model parameters all at once. Many of the model parameters cannot be reliably estimated from sparse data directly from patients, leading to identifiability issues (133). To overcome this, we may “pre-train” the models by gathering relevant parameter values and their reasonable ranges of variability from various experimental data and/or physical, biochemical, and biological reasoning. Pre-training can detour the difficulties in collecting all data modalities from every patient by utilizing partially matched multimodal datasets. We may obtain the correlation structures in the high-dimensional parameter space between parameters from model components describing different biological layers by aligning the corresponding partially paired data modalities. Recently emerging data linking genetic variations and cell-type- and/or condition-specific quantitative phenotypic variations can further help individualized model parameterizations (134–136). In most cases, the values can be constrained within a few orders of magnitude, within which the values can change either physically or pathologically. Then, we explore the plausible dynamical landscape of the multi-physiology models across the high-dimensional parameter space (**Figure 2**). Finally, we may calibrate the model to the patients undergoing immunotherapy of interest as an ensemble of parameter sets that recover observed or desired dynamical trajectories of immune behavior via approximate Bayesian computation (137). As a part of multi-physiology modeling, we should curate parameters for various biological and immunological processes and establish experimental platforms that facilitate accumulating parameter information (138).

To demonstrate how the multi-physiology modeling approach operates, we present a hypothetical scenario in which we treat a cold tumor to transform it into a hot tumor to make it more susceptible to T cell-targeting immunotherapies. First, single-cell and spatial transcriptomic data, along with clinical information, across tumors with varying immune phenotypes are collected. Multi-omics analysis enables the construction of a multiscale network representing intercellular and intracellular interactions in the tumor immune microenvironment. This network is then translated into mathematical equations via a reaction network scheme. We then form a multi-physiology model by implementing the equations into a multi-scale simulation framework combined with pharmacometric models for relevant immunotherapeutic agents. A tumor immune dynamical landscape is then constructed that maps high-dimensional model parameter space to tumor immune dynamics and clinical therapeutic outcomes by weaving existing data and massive simulations across the parameter space together. The landscape is then used to explore possible therapeutic outcomes under various therapeutic interventions to obtain insights into the transition between cold and hot tumors. Finally, by narrowing model parameters to reflect individual patient profiles, personalized strategies for precision immunotherapy are identified through individualized predictions.

## 6 Discussion

The advances in immunotherapeutics, such as antibody-based drugs, nanoparticle delivery vehicles, and adoptive cell therapies, are being accelerated in providing patients with new modes of treating immune-related diseases. At the same time, the exponential growth of multi-omics biological data, further accelerated by patient-derived experimental models, offers unprecedented insights into the immune system’s complexity (139). A number of studies have been conducted to relate the characteristics of patient-specific attributes to therapeutic outcomes in a data-driven manner (140, 141). However, we still lack a unified framework enabling predictive immunotherapies tailored to individual patients. In this article, we propose an overarching umbrella, “multi-physiology modeling” of the immune system. It quantitatively describes the immune system with its multiscale nonlinear dynamics of many interacting constituents and cellular phenotypic heterogeneities, together with PK/PD modeling that interfaces with individual patients. A major hurdle in achieving this is likely the lack of cross-disciplinary communications that resulted in discipline-oriented approaches, each limited.

With multi-physiology models of the immune system, what do we want to achieve eventually? First, we want to advance from mere statistical predictions of immunotherapeutic responses of predefined patient groups to quantitative and dynamic predictions of immunotherapeutic outcomes tailored to individual patients or at least more granular immune phenotypic groups. This will also allow more efficient drug target identification and virtual clinical trial platforms that perform combinatorial immunotherapeutic regimens. Clinicians who employ these platforms may collect a diseased tissue sample with relevant routine clinical data from the patient, which can then be transformed into more detailed immune profiling data. By conducting repeated simulations of the model under various immunotherapeutic scenarios, the clinician will be able to predict the outcomes of various treatment options and conclude the most suitable treatment method for the patient. Second, well-developed multi-physiology models will serve as integrative hubs to distill and accumulate vast amounts of immunological knowledge and data. This will accelerate not only our understanding of basic immunology related to immune-related diseases but also the efficiency and accuracy of clinical immunotherapeutics.

## Data Availability

The original contributions presented in the study are included in the article/supplementary material. Further inquiries can be directed to the corresponding author.

## References

[1] Dobosz P Dzieciatkowski T. The intriguing history of cancer immunotherapy. Front Immunol. (2019) 10:2965. doi: 10.3389/fimmu.2019.02965 31921205 PMC6928196

[2] Lee DSW Rojas OL Gommerman JL. B cell depletion therapies in autoimmune disease: advances and mechanistic insights. Nat Rev Drug Discov. (2021) 20:179–99. doi: 10.1038/s41573-020-00092-2 PMC7737718 33324003

[3] van de Veerdonk FL Giamarellos-Bourboulis E Pickkers P Derde L Leavis H Crevel van R. A guide to immunotherapy for COVID-19. Nat Med. (2022) 28:39–50. doi: 10.1038/s41591-021-01643-9 35064248

[4] Giamarellos-Bourboulis EJ Aschenbrenner AC Bauer M Bock C Calandra T Gat-Viks I. The pathophysiology of sepsis and precision-medicine-based immunotherapy. Nat Immunol. (2024) 25:19–28. doi: 10.1038/s41590-023-01660-5 38168953

[5] Marques L Costa B Pereira M Silva A Santos J Saldanha L. Advancing precision medicine: A review of innovative in silico approaches for drug development, clinical pharmacology and personalized healthcare. Pharmaceutics. (2024) 16. doi: 10.3390/pharmaceutics16030332 PMC10975777 38543226

[6] Ramos-Casals M Brahmer JR Callahan MK Flores-Chavez A Keegan N Khamashta MA. Immune-related adverse events of checkpoint inhibitors. Nat Rev Dis Primers. (2020) 6:38. doi: 10.1038/s41572-020-0160-6 32382051 PMC9728094

[7] Appelbaum J Price AE Oda K Zhang J Leung WH Tampella G. Drug-regulated CD33-targeted CAR T cells control AML using clinically optimized rapamycin dosing. J Clin Invest. (2024) 134. doi: 10.1172/JCI162593 PMC11060733 38502193

[8] Sancho-Araiz A Mangas-Sanjuan V Trocóniz IF. The role of mathematical models in immuno-oncology: challenges and future perspectives. Pharmaceutics. (2021) 13:1016–23. doi: 10.3390/pharmaceutics13071016 PMC8309057 34371708

[9] Mukherjee A Abraham S Singh A Balaji S Mukunthan KS. From data to cure: A comprehensive exploration of multi-omics data analysis for targeted therapies. Mol Biotechnol. (2025) 67:1269–89. doi: 10.1007/s12033-024-01133-6 PMC11928429 38565775

[10] Wong HS Park K Gola A Baptista AP Miller CH Deep D. A local regulatory T cell feedback circuit maintains immune homeostasis by pruning self-activated T cells. Cell. (2021) 184:3981–3997.e22. doi: 10.1016/j.cell.2021.05.028 34157301 PMC8390950

[11] Karki R Kanneganti TD. The ‘cytokine storm’: molecular mechanisms and therapeutic prospects. Trends Immunol. (2021) 42:681–705. doi: 10.1016/j.it.2021.06.001 34217595 PMC9310545

[12] Wang T Sun H Lu Z Jiang W Dai G Huang L. The CARDS toxin of Mycoplasma pneumoniae induces a positive feedback loop of type 1 immune response. Front Immunol. (2022) 13:1054788. doi: 10.3389/fimmu.2022.1054788 36532054 PMC9752573

[13] Jordan MB. Hemophagocytic lymphohistiocytosis: A disorder of T cell activation, immune regulation, and distinctive immunopathology. Immunol Rev. (2024) 322:339–50. doi: 10.1111/imr.v322.1 38100247

[14] Harlapur P Duddu AS Jolly MK. Dynamics of T-helper cell differentiation and plasticity: How have computational models improved our understanding? Curr Opin Syst Biol. (2024) 37. doi: 10.1016/j.coisb.2024.100508

[15] Derendorf H Schmidt S. Rowland and Tozer’s clinical pharmacokinetics and pharmacodynamics: concepts and applications. Philadelphia, PA, USA: Lippincott Williams & Wilkins (2019).

[16] Gabrielsson J Weiner D. Pharmacokinetic and pharmacodynamic data analysis: concepts and applications. Stockholm, Sweden: Swedish Pharmaceutical Press (2017).

[17] Owen JS Fiedler-Kelly J. Introduction to Population Pharmacokinetic/Pharmacodynamic Analysis with Nonlinear Mixed Effects Models. Hoboken, NJ, USA: Wiley (2014). 10.1038/psp.2014.51 PMC5684957 25531560

[18] Aghamiri SS Amin R Helikar T. Recent applications of quantitative systems pharmacology and machine learning models across diseases. J Pharmacokinet Pharmacodyn. (2022) 49:19–37. doi: 10.1007/s10928-021-09790-9 34671863 PMC8528185

[19] Qi T Liao X Cao Y. Development of bispecific T cell engagers: harnessing quantitative systems pharmacology. Trends Pharmacol Sci. (2023) 44:880–90. doi: 10.1016/j.tips.2023.09.009 PMC10843027 37852906

[20] Uatay A Gall L Irons L Tewari SG Zhu XS Gibbs M. Physiological indirect response model to omics-powered quantitative systems pharmacology model. J Pharm Sci. (2024) 113:11–21. doi: 10.1016/j.xphs.2023.10.032 37898164

[21] Zhang Y Zhang Z. The history and advances in cancer immunotherapy: understanding the characteristics of tumor-infiltrating immune cells and their therapeutic implications. Cell Mol Immunol. (2020) 17:807–21. doi: 10.1038/s41423-020-0488-6 PMC7395159 32612154

[22] Schett G Mackensen A Mougiakakos D. CAR T-cell therapy in autoimmune diseases. Lancet. (2023) 402:2034–44. doi: 10.1016/S0140-6736(23)01126-1 37748491

[23] Zhang J Huang Y Xi G Zhang F. HX008: a humanized PD-1 blocking antibody with potent antitumor activity and superior pharmacologic properties. mAbs. (2020) 12. doi: 10.1080/19420862.2020.1724751 PMC7153830 32106752

[24] Sharma P Goswami S Raychaudhuri D Siddiqui BA Singh P Nagarajan A. Immune checkpoint therapy-current perspectives and future directions. Cell. (2023) 186:1652–69. doi: 10.1016/j.cell.2023.03.006 37059068

[25] Luo H Wang W Mai J Yin R Cai X Li Q. The nexus of dynamic T cell states and immune checkpoint blockade therapy in the periphery and tumor microenvironment. Front Immunol. (2023) 14:1267918. doi: 10.3389/fimmu.2023.1267918 37881432 PMC10597640

[26] Takeuchi T. Cytokines and cytokine receptors as targets of immune-mediated inflammatory diseases-RA as a role model. Inflammation Regener. (2022) 42:35. doi: 10.1186/s41232-022-00221-x PMC9713106 36451227

[27] Yuan M Han Z Liang Y Sun Y He B Chen W. mRNA nanodelivery systems: targeting strategies and administration routes. Biomater Res. (2023) 27:90. doi: 10.1186/s40824-023-00425-3 37740246 PMC10517595

[28] Mirtaleb MS Falak R Heshmatnia J Bakhshandeh B Taheri RA Soleimanjahi H. An insight overview on COVID-19 mRNA vaccines: Advantageous, pharmacology, mechanism of action, and prospective considerations. Int Immunopharmacol. (2023) 117. doi: 10.1016/j.intimp.2023.109934 PMC9968612 36867924

[29] Verbeke R Hogan MJ Loré K Pardi N. Innate immune mechanisms of mRNA vaccines. Immunity. (2022) 55:1993–2005. doi: 10.1016/j.immuni.2022.10.014 36351374 PMC9641982

[30] Karim ME Haque ST Al-Busaidi H Bakhtiar A Tha KK Holl MMB. Scope and challenges of nanoparticle-based mRNA delivery in cancer treatment. Arch Pharm Res. (2022) 45:865–93. doi: 10.1007/s12272-022-01418-x PMC9686230 36422795

[31] Iyer VR P P Kaduskar BD Moharir SC Mishra RK. mRNA biotherapeutics landscape for rare genetic disorders. J Biosci. (2024) 49. doi: 10.1007/s12038-023-00415-6 38383978

[32] Sabbah M Jondreville L Lacan C Norol F Vieillard V Roos-Weil D. CAR-NK cells: A chimeric hope or a promising therapy? Cancers (Basel). (2022) 14. doi: 10.3390/cancers14153839 PMC9367380 35954502

[33] Maalej KM Merhi M Inchakalody VP Mestiri S Alam M Maccalli C. CAR-cell therapy in the era of solid tumor treatment: current challenges and emerging therapeutic advances. Mol Cancer. (2023) 22:20. doi: 10.1186/s12943-023-01723-z 36717905 PMC9885707

[34] Sterner RC Sterner RM. CAR-T cell therapy: current limitations and potential strategies. Blood Cancer J. (2021) 11:69. doi: 10.1038/s41408-021-00459-7 33824268 PMC8024391

[35] Yang J Zhou W Li D Niu T Wang W. BCMA-targeting chimeric antigen receptor T-cell therapy for multiple myeloma. Cancer Lett. (2023) 553. doi: 10.1016/j.canlet.2022.215949 36216149

[36] Xiao BF Zhang JT Zhu YG Cui XR Lu ZM Yu BT. Chimeric antigen receptor T-cell therapy in lung cancer: potential and challenges. Front Immunol. (2021) 12:782775. doi: 10.3389/fimmu.2021.782775 34790207 PMC8591168

[37] Riet T Chmielewski M. Regulatory CAR-T cells in autoimmune diseases: Progress and current challenges. Front Immunol. (2022) 13:934343. doi: 10.3389/fimmu.2022.934343 36032080 PMC9399761

[38] Baeten P Van Zeebroeck L Kleinewietfeld M Hellings N Broux B. Improving the efficacy of regulatory T cell therapy. Clin Rev Allergy Immunol. (2022) 62:363–81. doi: 10.1007/s12016-021-08866-1 PMC8256646 34224053

[39] Kleef R Dank M Herold M Agoston EI Lohinszky J Martinek E. Comparison of the effectiveness of integrative immunomodulatory treatments and conventional therapies on the survival of selected gastrointestinal cancer patients. Sci Rep. (2023) 13. doi: 10.1038/s41598-023-47802-5 PMC10663566 37990076

[40] Tyagi P Hafron J Kaufman J Chancellor M. Enhancing therapeutic efficacy and safety of immune checkpoint inhibition for bladder cancer: A comparative analysis of injectable vs. Intravesical administration. Int J Mol Sci. (2024) 25. doi: 10.3390/ijms25094945 PMC11084450 38732167

[41] Bell HN Zou W. Beyond the barrier: unraveling the mechanisms of immunotherapy resistance. Annu Rev Immunol. (2024) 42:521–50. doi: 10.1146/annurev-immunol-101819-024752 PMC11213679 38382538

[42] Ryman JT Meibohm B. Pharmacokinetics of monoclonal antibodies. CPT: Pharmacometrics Syst Pharmacol. (2017) 6:576–88. doi: 10.1002/psp4.12224 PMC5613179 28653357

[43] Kamath AV. Translational pharmacokinetics and pharmacodynamics of monoclonal antibodies. Drug Discov Today. (2016) 21-22:75–83. doi: 10.1016/j.ddtec.2016.09.004 27978991

[44] Friedrich SW Linz SC Stoll BR Baxter LT Munn LL Jain RK. Antibody-directed effector cell therapy of tumors: analysis and optimization using a physiologically based pharmacokinetic model. Neoplasia. (2002) 4:449–63. doi: 10.1038/sj.neo.7900260 PMC1661679 12192604

[45] Zheng S Niu J Geist B Fink D Xu Z Zhou H. A minimal physiologically based pharmacokinetic model to characterize colon TNF suppression and treatment effects of an anti-TNF monoclonal antibody in a mouse inflammatory bowel disease model. mAbs. (2020) 12. doi: 10.1080/19420862.2020.1813962 PMC7531524 32967523

[46] Yan T Yu L Shangguan D Li W Liu N Chen Y. Advances in pharmacokinetics and pharmacodynamics of PD-1/PD-L1 inhibitors. Int Immunopharmacol. (2023) 115. doi: 10.1016/j.intimp.2022.109638 36587500

[47] Attarwala H Lumley M Liang M Ivaturi V Senn J. Translational pharmacokinetic/pharmacodynamic model for mRNA-3927, an investigational therapeutic for the treatment of propionic acidemia. Nucleic Acid Ther. (2023) 33:141–7. doi: 10.1089/nat.2022.0036 PMC10066765 36577040

[48] Naasani I. Establishing the pharmacokinetics of genetic vaccines is essential for maximising their safety and efficacy. Clin Pharmacokinetics. (2022) 61:921–7. doi: 10.1007/s40262-022-01149-8 35821373

[49] Selvaggio G Leonardelli L Lofano G Fresnay S Parolo S Medini D. A quantitative systems pharmacology approach to support mRNA vaccine development and optimization. CPT: Pharmacometrics Syst Pharmacol. (2021) 10:1448–51. doi: 10.1002/psp4.v10.12 PMC8674002 34672423

[50] Kutumova EO Akberdin IR Kiselev IN Sharipov RN Egorova VS Syrocheva AO. Physiologically based pharmacokinetic modeling of nanoparticle biodistribution: A review of existing models, simulation software, and data analysis tools. Int J Mol Sci. (2022) 23. doi: 10.3390/ijms232012560 PMC9604366 36293410

[51] Kirouac DC Zmurchok C Morris D. Making drugs from T cells: The quantitative pharmacology of engineered T cell therapeutics. NPJ Syst Biol Appl. (2024) 10. doi: 10.1038/s41540-024-00355-3 PMC10948391 38499572

[52] Singh AP Zheng X Lin-Schmidt X Chen W Carpenter TJ Zong A. Development of a quantitative relationship between CAR-affinity, antigen abundance, tumor cell depletion and CAR-T cell expansion using a multiscale systems PK-PD model. mAbs. (2019) 12. doi: 10.1080/19420862.2019.1688616 PMC6927769 31852337

[53] Nukala U Rodriguez Messan M Yogurtcu ON Wang X Yang H. A systematic review of the efforts and hindrances of modeling and simulation of CAR T-cell therapy. AAPS J. (2021) 23. doi: 10.1208/s12248-021-00579-9 33835308

[54] Singh AP Chen W Zheng X Mody H Carpenter TJ Zong A. Bench-to-bedside translation of chimeric antigen receptor (CAR) T cells using a multiscale systems pharmacokinetic-pharmacodynamic model: A case study with anti-BCMA CAR-T. CPT: Pharmacometrics Syst Pharmacol. (2021) 10:362–76. doi: 10.1002/psp4.12598 PMC8099446 33565700

[55] Qi T McGrath K Ranganathan R Dotti G Cao Y. Cellular kinetics: A clinical and computational review of CAR-T cell pharmacology. Advanced Drug Delivery Rev. (2022) 188. doi: 10.1016/j.addr.2022.114421 PMC9520251 35809868

[56] Chelliah V Lazarou G Bhatnagar S Gibbs JP Nijsen M Ray A. Quantitative systems pharmacology approaches for immuno-oncology: adding virtual patients to the development paradigm. Clin Pharmacol Ther. (2021) 109:605–18. doi: 10.1002/cpt.v109.3 PMC7983940 32686076

[57] Kumar R Thiagarajan K Jagannathan L Liu L Mayawala K de Alwis D. Beyond the single average tumor: Understanding IO combinations using a clinical QSP model that incorporates heterogeneity in patient response. CPT Pharmacometrics Syst Pharmacol. (2021) 10:684–95. doi: 10.1002/psp4.12637 PMC8302246 33938166

[58] Lazarou G Chelliah V Small BG Walker M van der Graaf PH Kierzek AM. Integration of omics data sources to inform mechanistic modeling of immune-oncology therapies: A tutorial for clinical pharmacologists. Clin Pharmacol Ther. (2020) 107:858–70. doi: 10.1002/cpt.v107.4 PMC7158209 31955413

[59] Arulraj T Wang H Emens LA Santa-Maria CA Popel AS. A transcriptome-informed QSP model of metastatic triple-negative breast cancer identifies predictive biomarkers for PD-1 inhibition. Sci Adv. (2023) 9. doi: 10.1126/sciadv.adg0289 PMC10313177 37390206

[60] Mody H Ogasawara K Zhu X Miles D Shastri PN Gokemeijer J. Best practices and considerations for clinical pharmacology and pharmacometric aspects for optimal development of CAR-T and TCR-T cell therapies: an industry perspective. Clin Pharmacol Ther. (2023) 114:530–57. doi: 10.1002/cpt.v114.3 37393588

[61] Martinez-Hernandez R Sanchez de la Blanca N Sacristan-Gomez P Serrano-Somavilla A Munoz De Nova JL Sanchez Cabo F. Unraveling the molecular architecture of autoimmune thyroid diseases at spatial resolution. Nat Commun. (2024) 15. doi: 10.1038/s41467-024-50192-5 PMC11246508 39003267

[62] Tripathi S Tsang JS Park K. Systems immunology of regulatory T cells: can one circuit explain it all? Trends Immunol. (2023) 44:766–81. doi: 10.1016/j.it.2023.08.007 PMC10543564 37690962

[63] Germain RN. The art of the probable: system control in the adaptive immune system. Science. (2001) 293:240–5. doi: 10.1126/science.1062946 11452112

[64] Simeonov DR Park K Cortez JT Young A Li Z Nguyen V. Non-coding sequence variation reveals fragility within interleukin 2 feedback circuitry and shapes autoimmune disease risk. bioRxiv. (2023). doi: 10.1101/2023.06.17.545426v1

[65] Pankiw M Brezden-Masley C Charames GS. Comprehensive genomic profiling for oncological advancements by precision medicine. Med Oncol. (2023) 41. doi: 10.1007/s12032-023-02228-x 37993657

[66] Mogilenko DA Shchukina I Artyomov MN. Immune ageing at single-cell resolution. Nat Rev Immunol. (2022) 22:484–98. doi: 10.1038/s41577-021-00646-4 PMC8609266 34815556

[67] Dyikanov D Zaitsev A Vasileva T Wang I Sokolov AA Bolshakov ES. Comprehensive peripheral blood immunoprofiling reveals five immunotypes with immunotherapy response characteristics in patients with cancer. Cancer Cell. (2024) 42:759–779.e12. doi: 10.1016/j.ccell.2024.04.008 38744245

[68] Feng X Tonon L Li H Darbo E Pleasance E Macagno N. Comprehensive immune profiling unveils a subset of leiomyosarcoma with “Hot” Tumor immune microenvironment. Cancers (Basel). (2023) 15. doi: 10.3390/cancers15143705 PMC10378143 37509366

[69] Davis MM. Systems immunology. Curr Opin Immunol. (2020) 65:79–82. doi: 10.1016/j.coi.2020.06.006 32738786 PMC7390724

[70] Germain RN Meier-Schellersheim M Nita-Lazar A Fraser ID. Systems biology in immunology: a computational modeling perspective. Annu Rev Immunol. (2011) 29:527–85. doi: 10.1146/annurev-immunol-030409-101317 PMC3164774 21219182

[71] Tsang JS. Utilizing population variation, vaccination, and systems biology to study human immunology. Trends Immunol. (2015) 36:479–93. doi: 10.1016/j.it.2015.06.005 PMC4979540 26187853

[72] Cui A Huang T Li S Ma A Perez JL Sander C. Dictionary of immune responses to cytokines at single-cell resolution. Nature. (2024) 625:377–84. doi: 10.1038/s41586-023-06816-9 PMC10781646 38057668

[73] Schafer PSL Dimitrov D Villablanca EJ Saez-Rodriguez J. Integrating single-cell multi-omics and prior biological knowledge for a functional characterization of the immune system. Nat Immunol. (2024) 25:405–17. doi: 10.1038/s41590-024-01768-2 38413722

[74] Wen S Mo S Zhou J Lv Y Khazaie K Yu G. Editorial: Single-cell and spatial-omics in delineating immune-related diseases. Front Cell Dev Biol. (2024) 12:1365242. doi: 10.3389/fcell.2024.1365242 38298217 PMC10829097

[75] Melms JC Biermann J Huang H Wang Y Nair A Tagore S. A molecular single-cell lung atlas of lethal COVID-19. Nature. (2021) 595:114–9. doi: 10.1038/s41586-021-03569-1 PMC8814825 33915568

[76] Hwang B Lee JH Bang D. Single-cell RNA sequencing technologies and bioinformatics pipelines. Exp Mol Med. (2018) 50:1–14. doi: 10.1038/s12276-018-0071-8 PMC6082860 30089861

[77] Kolodziejczyk AA Kim JK Svensson V Marioni JC Teichmann SA. The technology and biology of single-cell RNA sequencing. Mol Cell. (2015) 58:610–20. doi: 10.1016/j.molcel.2015.04.005 26000846

[78] Sparks R Lau WW Liu C Han KL Vrindten KL Sun G. Influenza vaccination reveals sex dimorphic imprints of prior mild COVID-19. Nature. (2023) 614:752–61. doi: 10.1038/s41586-022-05670-5 PMC10481789 36599369

[79] Albarnaz JD Weekes MP. Proteomic analysis of antiviral innate immunity. Curr Opin Virol. (2023) 58. doi: 10.1016/j.coviro.2022.101291 36529073

[80] Preissl S Gaulton KJ Ren B. Characterizing cis-regulatory elements using single-cell epigenomics. Nat Rev Genet. (2023) 24:21–43. doi: 10.1038/s41576-022-00509-1 35840754 PMC9771884

[81] Chen C Wang J Pan D Wang X Xu Y Yan J. Applications of multi-omics analysis in human diseases. MedComm (2020). (2023) 4. doi: 10.1002/mco2.315 PMC10390758 37533767

[82] Vandereyken K Sifrim A Thienpont B Voet T. Methods and applications for single-cell and spatial multi-omics. Nat Rev Genet. (2023) 24:494–515. doi: 10.1038/s41576-023-00580-2 36864178 PMC9979144

[83] Lakkis J Schroeder A Su K Lee MYY Bashore AC Reilly MP. A multi-use deep learning method for CITE-seq and single-cell RNA-seq data integration with cell surface protein prediction and imputation. Nat Mach Intell. (2022) 4:940–52. doi: 10.1038/s42256-022-00545-w PMC9979929 36873621

[84] Zhang X Song B Carlino MJ Li G Ferchen K Chen M. An immunophenotype-coupled transcriptomic atlas of human hematopoietic progenitors. Nat Immunol. (2024) 25:703–15. doi: 10.1038/s41590-024-01782-4 PMC11003869 38514887

[85] Cheong JG Ravishankar A Sharma S Parkhurst CN Grassmann SA Wingert CK. Epigenetic memory of coronavirus infection in innate immune cells and their progenitors. Cell. (2023) 186:3882–3902.e24. doi: 10.1016/j.cell.2023.07.019 37597510 PMC10638861

[86] Hao Y Stuart T Kowalski MH Choudhary S Hoffman P Hartman A. Dictionary learning for integrative, multimodal and scalable single-cell analysis. Nat Biotechnol. (2024) 42:293–304. doi: 10.1038/s41587-023-01767-y 37231261 PMC10928517

[87] Lin JR Wang S Coy S Chen YA Yapp C Tyler M. Multiplexed 3D atlas of state transitions and immune interaction in colorectal cancer. Cell. (2023) 186:363–381.e19. doi: 10.1016/j.cell.2022.12.028 36669472 PMC10019067

[88] Lewis SM Asselin-Labat ML Nguyen Q Berthelet J Tan X Wimmer VC. Spatial omics and multiplexed imaging to explore cancer biology. Nat Methods. (2021) 18:997–1012. doi: 10.1038/s41592-021-01203-6 34341583

[89] Liu Y DiStasio M Su G Asashima H Enninful A Qin X. High-plex protein and whole transcriptome co-mapping at cellular resolution with spatial CITE-seq. Nat Biotechnol. (2023) 41:1405–9. doi: 10.1038/s41587-023-01676-0 PMC10567548 36823353

[90] Eftimie R Gillard JJ Cantrell DA. Mathematical models for immunology: current state of the art and future research directions. Bull Math Biol. (2016) 78:2091–134. doi: 10.1007/s11538-016-0214-9 PMC5069344 27714570

[91] Cappuccio A Tieri P Castiglione F. Multiscale modelling in immunology: a review. Briefings Bioinf. (2016) 17:408–18. doi: 10.1093/bib/bbv012 25810307

[92] Smith AM. Decoding immune kinetics: unveiling secrets using custom-built mathematical models. Nat Methods. (2024) 21:744–7. doi: 10.1038/s41592-024-02265-y PMC11488966 38710785

[93] Creemers JHA Textor J. Leveraging mathematical models to improve the statistical robustness of cancer immunotherapy trials. Curr Opin Syst Biol. (2025) 40. doi: 10.1016/j.coisb.2024.100540

[94] Bonin CRB Fernandes GC Dos Santos RW Lobosco M. A qualitatively validated mathematical-computational model of the immune response to the yellow fever vaccine. BMC Immunol. (2018) 19. doi: 10.1186/s12865-018-0252-1 PMC5970533 29801432

[95] Yiu HH Graham AL Stengel RF. Dynamics of a cytokine storm. PLoS One. (2012) 7. doi: 10.1371/journal.pone.0045027 PMC3462188 23049677

[96] Grebennikov DS Donets DO Orlova OG Argilaguet J Meyerhans A Bocharov GA. Mathematical modeling of the intracellular regulation of immune processes. Mol Biol. (2019) 53:718–31. doi: 10.1134/S002689331905008X 31661480

[97] Caudill L Lynch F. A mathematical model of the inflammatory response to pathogen challenge. Bull Math Biol. (2018) 80:2242–71. doi: 10.1007/s11538-018-0459-6 29951890

[98] Smith AM. Validated models of immune response to virus infection. Curr Opin Syst Biol. (2018) 12:46–52. doi: 10.1016/j.coisb.2018.10.005 31723715 PMC6853615

[99] Leon C Tokarev A Bouchnita A Volpert V. Modelling of the innate and adaptive immune response to SARS viral infection, cytokine storm and vaccination. Vaccines (Basel). (2023) 11. doi: 10.3390/vaccines11010127 PMC9861811 36679972

[100] Pigozzo AB Macedo GC Santos RW Lobosco M. On the computational modeling of the innate immune system. BMC Bioinf. (2013) 14 Suppl 6. doi: 10.1186/1471-2105-14-S6-S7 PMC3633047 23734602

[101] Bocharov G Meyerhans A Bessonov N Trofimchuk S Volpertf V. Modelling the dynamics of virus infection and immune response in space and time. Int J Parallel Emergent Distributed Syst. (2016) 34(4):341–55. doi: 10.1080/17445760

[102] Sardar M Khajanchi S Biswas S. Stochastic dynamics of a nonlinear tumor-immune competitive system. Nonlinear Dynamics. (2025) 4395–423. doi: 10.1007/s11071-024-09768-5

[103] Figueroa-Morales N Leon K Mulet R. Stochastic approximation to the T cell mediated specific response of the immune system. J Theor Biol. (2012) 295:37–46. doi: 10.1016/j.jtbi.2011.11.003 22100422

[104] Graw F Perelson AS. Modeling viral spread. Annu Rev Virol. (2016) 3:555–72. doi: 10.1146/annurev-virology-110615-042249 PMC5072357 27618637

[105] Tong X Chen J Miao H Li T Zhang L. Development of an agent-based model (ABM) to simulate the immune system and integration of a regression method to estimate the key ABM parameters by fitting the experimental data. PLoS One. (2015) 10. doi: 10.1371/journal.pone.0141295 PMC4633145 26535589

[106] Shi Z. An agent-based model of a hepatic inflammatory response to salmonella: A computational study under a large set of experimental data. PLoS One. (2016) 11. doi: 10.1371/journal.pone.0161131 PMC4996536 27556404

[107] Petrucciani A Hoerter A Kotze L Plessis ND Pienaar E. In silico agent-based modeling approach to characterize multiple *in vitro* tuberculosis 2 infection models. (2023). doi: 10.1101/2023.03.13.532338v1 PMC10959380 38517920

[108] Ma C Gurkan-Cavusoglu E. A comprehensive review of computational cell cycle models in guiding cancer treatment strategies. NPJ Syst Biol Appl. (2024) 10:71. doi: 10.1038/s41540-024-00397-7 38969664 PMC11226463

[109] Agmon E Spangler RK Skalnik CJ Poole W Peirce SM Morrison JH. Vivarium: an interface and engine for integrative multiscale modeling in computational biology. Bioinformatics. (2022) 38:1972–9. doi: 10.1093/bioinformatics/btac049 PMC8963310 35134830

[110] Castiglione F Bernaschi M. C-ImmSim∗: playing with the immune response. In: Sixteenth International Symposium on Mathematical Theory of Networks and Systems. Katholieke Universiteit Leuven, Belgium (2004).

[111] Handel A. A software package for immunologists to learn simulation modeling. BMC Immunol. (2020) 21:1. doi: 10.1186/s12865-019-0321-0 31898481 PMC6941246

[112] Fidler M Wilkins JJ Hooijmaijers R Post TM Schoemaker R Trame MN. Nonlinear mixed-effects model development and simulation using nlmixr and related R open-source packages. CPT Pharmacometrics Syst Pharmacol. (2019) 8:621–33. doi: 10.1002/psp4.12445 PMC6765694 31207186

[113] NONMEM. Available online at: https://www.iconplc.com/solutions/technologies/nonmem (Accessed May 20, 2025).

[114] Simmune Project. Available online at: https://www.niaid.nih.gov/research/simmune-project (Accessed May 20, 2025).

[115] COPASI. Available online at: https://copasi.org/Research/ (Accessed May 20, 2025).

[116] CompuCell3D. Available online at: https://compucell3d.org/ (Accessed May 20, 2025).

[117] NMsim. Available online at: https://nmautoverse.github.io/NMsim/ (Accessed May 20, 2025).

[118] Pharmpy. Available online at: https://pharmpy.github.io/latest/index.html (Accessed May 20, 2025).

[119] Weaver JJA Smith AM. Quantitatively mapping immune control during influenza. Curr Opin Syst Biol. (2024) 38. doi: 10.1016/j.coisb.2024.100516 PMC11488648 39430368

[120] Scharf S Ackermann J Wurzel P Hansmann M-L Koch I. Computational systems biology of cellular processes in the human lymph node. Curr Opin Syst Biol. (2024) 38. doi: 10.1016/j.coisb.2024.100518

[121] Keyes DE McInnes LC Woodward C Gropp W Myra E Pernice M. Multiphysics simulations: challenges and opportunities. Int J High Performance Computing Appl. (2013) 27:4–83. doi: 10.1177/1094342012468181

[122] Germain RN. Will systems biology deliver its promise and contribute to the development of new or improved vaccines? What really constitutes the study of “Systems biology” and how might such an approach facilitate vaccine design. Cold Spring Harb Perspect Biol. (2018) 10. doi: 10.1101/cshperspect.a033308 PMC6071490 29038120

[123] Troule K Petryszak R Cakir B Cranley J Harasty A Prete M. CellPhoneDB v5: inferring cell-cell communication from single-cell multiomics data. Nat Protoc. (2025). doi: 10.1038/s41596-024-01137-1 40133495

[124] Jin S Guerrero-Juarez CF Zhang L Chang I Ramos R Kuan CH. Inference and analysis of cell-cell communication using CellChat. Nat Commun. (2021) 12. doi: 10.1038/s41467-021-21246-9 PMC7889871 33597522

[125] Dimitrov D Turei D Garrido-Rodriguez M Burmedi PL Nagai JS Boys C. Comparison of methods and resources for cell-cell communication inference from single-cell RNA-Seq data. Nat Commun. (2022) 13. doi: 10.1038/s41467-022-30755-0 PMC9184522 35680885

[126] Turei D Korcsmaros T Saez-Rodriguez J. OmniPath: guidelines and gateway for literature-curated signaling pathway resources. Nat Methods. (2016) 13:966–7. doi: 10.1038/nmeth.4077 27898060

[127] Bravo Gonzalez-Blas C De Winter S Hulselmans G Hecker N Matetovici I Christiaens V. SCENIC+: single-cell multiomic inference of enhancers and gene regulatory networks. Nat Methods. (2023) 20. doi: 10.1038/s41592-023-01938-4 PMC10482700 37443338

[128] Muller-Dott S Tsirvouli E Vazquez M Ramirez Flores RO Badia IMP Fallegger R. Expanding the coverage of regulons from high-confidence prior knowledge for accurate estimation of transcription factor activities. Nucleic Acids Res. (2023) 51:10934–49. doi: 10.1093/nar/gkad841 PMC10639077 37843125

[129] Browaeys R Saelens W Saeys Y. NicheNet: modeling intercellular communication by linking ligands to target genes. Nat Methods. (2020) 17:159–62. doi: 10.1038/s41592-019-0667-5 31819264

[130] Cheemalavagu N Shoger KE Cao YM Michalides BA Botta SA Faeder JR. Predicting gene-level sensitivity to JAK-STAT signaling perturbation using a mechanistic-to-machine learning framework. Cell Syst. (2024) 15:37–48.e4. doi: 10.1016/j.cels.2023.12.006 38198893 PMC10812086

[131] Arce MM Umhoefer JM Arang N Kasinathan S Freimer JW Steinhart Z. Central control of dynamic gene circuits governs T cell rest and activation. Nature. (2025) 637:930–9. doi: 10.1038/s41586-024-08314-y PMC11754113 39663454

[132] Park K Pruestel T Lu Y Tsang JS. Machine learning of stochastic gene network phenotypes. (2019). doi: 10.1101/825943v1

[133] Steinheuer LM Kluemper N Bald T Thurley K. Untangling cell–cell communication networks and on-treatment response in immunotherapy. Curr Opin Syst Biol. (2025) 40. doi: 10.1016/j.coisb.2024.100534

[134] Farh KK Marson A Zhu J Kleinewietfeld M Housley WJ Beik S. Genetic and epigenetic fine mapping of causal autoimmune disease variants. Nature. (2015) 518:337–43. doi: 10.1038/nature13835 PMC4336207 25363779

[135] Simeonov DR Gowen BG Boontanrart M Roth TL Gagnon JD Mumbach MR. Discovery of stimulation-responsive immune enhancers with CRISPR activation. Nature. (2017) 549:111–5. doi: 10.1038/nature23875 PMC5675716 28854172

[136] Soskic B Cano-Gamez E Smyth DJ Ambridge K Ke Z Matte JC. Immune disease risk variants regulate gene expression dynamics during CD4(+) T cell activation. Nat Genet. (2022) 54:817–26. doi: 10.1038/s41588-022-01066-3 PMC9197762 35618845

[137] Anderson HG Takacs GP Harris DC Kuang Y Harrison JK Stepien TL. Global stability and parameter analysis reinforce therapeutic targets of PD-L1-PD-1 and MDSCs for glioblastoma. J Math Biol. (2023) 88. doi: 10.1007/s00285-023-02027-y PMC10724342 38099947

[138] Sender R Weiss Y Navon Y Milo I Azulay N Keren L. The total mass, number, and distribution of immune cells in the human body. Proc Natl Acad Sci U S A. (2023) 120. doi: 10.1073/pnas.2308511120 PMC10623016 37871201

[139] Mackenzie NJ Nicholls C Templeton AR Perera MP Jeffery PL Zimmermann K. Modelling the tumor immune microenvironment for precision immunotherapy. Clin Transl Immunol. (2022) 11:e1400. doi: 10.1002/cti2.v11.6 PMC9234475 35782339

[140] Fan L Li M Zhou X Jia X Tian H Wen Q. T cell-related circRNA pairs to predict prognosis of patients with esophageal squamous cell carcinoma. Int Immunopharmacol. (2024) 141:112909. doi: 10.1016/j.intimp.2024.112909 39154531

[141] Qiu Q Xing L Wang Y Feng A Wen Q. Development and validation of a radiomics nomogram using computed tomography for differentiating immune checkpoint inhibitor-related pneumonitis from radiation pneumonitis for patients with non-small cell lung cancer. Front Immunol. (2022) 13:870842. doi: 10.3389/fimmu.2022.870842 35558076 PMC9088878

